# IGF2 Ameliorates Amyloidosis, Increases Cholinergic Marker Expression and Raises BMP9 and Neurotrophin Levels in the Hippocampus of the APPswePS1dE9 Alzheimer’s Disease Model Mice

**DOI:** 10.1371/journal.pone.0094287

**Published:** 2014-04-14

**Authors:** Tiffany J. Mellott, Sarah M. Pender, Rebecca M. Burke, Erika A. Langley, Jan Krzysztof Blusztajn

**Affiliations:** Department of Pathology and Laboratory Medicine, Boston University School of Medicine, Boston, Massachusetts, United States of America; University of Leipzig, Germany

## Abstract

The development of an effective therapy for Alzheimer’s disease (AD) is a major challenge to biomedical sciences. Because much of early AD pathophysiology includes hippocampal abnormalities, a viable treatment strategy might be to use trophic factors that support hippocampal integrity and function. IGF2 is an attractive candidate as it acts in the hippocampus to enhance memory consolidation, stimulate adult neurogenesis and upregulate cholinergic marker expression and acetylcholine (ACh) release. We performed a seven-day intracerebroventricular infusion of IGF2 in transgenic APPswe.PS1dE9 AD model mice that express green fluorescent protein in cholinergic neurons (APP.PS1/CHGFP) and in wild type WT/CHGFP littermates at 6 months of age representing early AD-like disease. IGF2 reduced the number of hippocampal Aβ40- and Aβ42-positive amyloid plaques in APP.PS1/CHGFP mice. Moreover, IGF2 increased hippocampal protein levels of the ACh-synthesizing enzyme, choline acetyltransferase in both WT/CHGFP and APP.PS1/CHGFP mice. The latter effect was likely mediated by increased protein expression of the cholinergic differentiating factor, BMP9, observed in IGF2-treated mice as compared to controls. IGF2 also increased the protein levels of hippocampal NGF, BDNF, NT3 and IGF1 and of doublecortin, a marker of neurogenesis. These data show that IGF2 administration is effective in reversing and preventing several pathophysiologic processes associated with AD and suggest that IGF2 may constitute a therapeutic target for AD.

## Introduction

Alzheimer’s disease (AD) pathology is characterized by cerebral accumulation of extracellular amyloid, intraneuronal neurofibrillary tangles, neurotransmitter abnormalities and, ultimately, loss of synapses and neuronal death. The amyloid is composed of Aβ peptides that are toxic to neurons [1]. Among the numerous neurotransmitter abnormalities in brains of AD patients, the cholinergic defect – thought to contribute to the amnesia that is so prominent in this illness [2]–[4] – is the result of the degeneration and/or malfunction of the basal forebrain cholinergic neurons (BFCN) [3]. BFCN are vulnerable to Aβ toxicity and we [5] and others [6]–[8] showed that Aβ impairs BFCN function in the absence of cell death. Thus, reduction of Aβ accumulation and generation of a trophic environment for BFCN are rational objectives in designing an AD therapy. We tested this idea using insulin-like growth factor 2 (IGF2) as a therapeutic agent based on studies showing that IGF2 mRNA levels decline in the frontal cortex of AD patients at relatively early stages of neuropathology (Braak and Braak 2–3) [9] and on a growing body of data showing beneficial effects of IGF2 on neural function. Intrahippocampal injections of IGF2 in rats [10], [11] and mice [12], [13] enhanced memory function, while antagonizing the action of endogenous IGF2 impaired memory [10], [12], [14] indicating the role of the locally-produced IGF2 in this process. IGF2 is expressed in the subgranular zone of the dentate gyrus, and endogenous IGF2 upregulates the proliferation of neural stem cells in this brain region [15] and, moreover, intrahippocampal injections of IGF2 promote the survival of adult-born neurons in the dentate granule cell layer [12], [13]. IGF2 increases the release of acetylcholine (ACh) from BFCN [16]–[18] and prevents the Aβ-evoked neurotoxicity in cultured septal neurons [19] and in hippocampal cultures [20].

We used the APPswe/PS1deltaE9 (APP.PS1) transgenic mice as a model of AD [21], [22]. To facilitate studies of cholinergic neurons, we crossed these mice with a transgenic strain that expresses the enhanced green fluorescent protein (eGFP) specifically in cholinergic cells [23], [24]. We infused these cholinergic neuron eGFP-expressing AD model mice intracerebroventriculary (icv) with IGF2 for 7 days. This treatment reduced the hippocampal amyloid plaque number, increased protein expression of the ACh–synthesizing enzyme, choline acetyltransferase (CHAT), and the levels of a cholinergic differentiating factor, BMP9, and of trophic factors NGF, BDNF, NT3 and IGF1, as well as a marker of neurogenesis, doublecortin (DCX), indicating that IGF2 exhibits efficacy as an AD treatment in this model.

## Materials and Methods

### Ethics Statement

All animal procedures were performed in accordance with the Animal Welfare Act (Animal Welfare Assurance Number A-3316-01) and the principles of the NIH Guide for the Care and Use of Laboratory Animals and were approved by the Institutional Animal Care and Use Committee of Boston University. All surgical procedures were performed under isoflurane anesthesia, and all efforts were made to minimize suffering.

### Animals and Surgical Procedures

We used the APPswe/PS1deltaE9 (APP.PS1) mice that express murine amyloid precursor protein (APP) with the human Aβ amino acid sequence harboring mutations that cause a familial form of AD (the Swedish mutation APP(K595N/M596L; APPswe) and a mutated form of presenilin 1 (PS1 with exon 9 deleted; PS1dE9) [21]. Although no model of AD fully recapitulates the human disease [25], APP.PS1 mice are well suited for our studies because they exhibit: 1) high production of Aβ peptides in brain and accumulation of amyloid plaques by 4–6 months of age [22], 2) cholinergic defects [26]–[31], and 3) cognitive impairments [31]–[36]. We crossed these mice with a transgenic CHGFP strain that expresses the enhanced green fluorescent protein (eGFP) specifically in cholinergic cells [23], [24]. Thus, our studies were performed on mice that either did not express the AD-related transgenes, designated WT/CHGFP, or their AD model littermates designated APP.PS1/CHGFP. We studied animals at 6 months of age when the APP.PS1 mice exhibit rapid rate of amyloid accumulation [37] and have normal CHAT activity, although they show some impairment of the BFCN projections [26]. This time point represents early, rapidly progressing, stage of pathogenesis. Homozygous CHGFP (B6.Cg-Tg(RP23-268L19-EGFP)2Mik/J) [23], [24] females were crossed to hemizygous APP.PS1 (B6C3-Tg(APPswe,PSEN1dE9)85Dbo/Mmjax) [22], [26] males to generate WT/CHGFP and APP.PS1/CHGFP experimental subjects.

We performed intraventricular infusion of vehicle or 50 ng/h of human recombinant IGF2 (Peprotech) for 7 days. An equal number of male and female mice at 6 months of age (n = 6 per group, 4 groups: PBS WT/CHGFP, PBS APP.PS1/CHGFP, IGF2 WT/CHGFP, IGF2 APP.PS1/CHGFP) were anesthetized using 5% isoflurane in oxygen delivered at a rate of 2.5 L/min. Following induction, isoflurane was given at 2.5% to maintain anesthesia. During the induction phase, mice received 0.04 mg/kg of buprenex analgesic. All surgical steps were performed aseptically on a sterile field. A midsagittal incision was made on the scalp and a subcutaneous tunnel was opened between the shoulder blades, where the Alzet osmotic pumps (model 1002; pumping rate 0.25 µl/h) were implanted. The stereotactic apparatus was used to position the cannula connected to the pump in the lateral ventricle through a small hole in the skull at the following coordinates relative to bregma: posterior: −0.6 mm, lateral: −1.2 mm, depth: −2.0 mm. The cannula was fixed in place with Loctite adhesive (Alzet) and dental cement. The mice received prophylactic antibiotic (ampicillin 35 mg/kg, s.c.) and 1Qml of 0.9% sterile saline s.c. for hydration purposes. The incision was closed with silk sutures and dabbed with Vetbond (3 M). The mice recovered from anesthesia breathing pure oxygen at a rate of 2.5 L/min for approximately 1 min. Following surgery, the mice were placed on a heating pad and subsequently back in their home cages. Animals received buprenex (0.04 mg/kg, s.c.) every 12 h post-operatively for 2 days. They were inspected daily for signs of distress and wound healing was monitored. After 7 days, the mice were killed by CO_2_ inhalation and their brains rapidly dissected on ice.

### ElISA

The hippocampus was homogenized in a lysis buffer containing 0.05 M Tris-HCl pH 7.5, 0.15 M NaCl, 1% NP-40, 1 mM Na-orthovanadate, 0.001% sodium fluoride, 1% protease inhibitor cocktail (Sigma). BDNF, NT3, and NGF were assayed using the Emax® immunoassay system (Promega) and FGF2 and IGF1 were assayed using the Quantikine® sandwich ELISA kit (R&D Systems) according to manufacturer’s instructions and as described in our previous publications [38]–[41]. Aβ40 and Aβ42 levels were assayed using Aβ40 Human ELISA Kit and Aβ42 Human ELISA Kit according to the manufacturer’s instructions (Invitrogen).

### Immunoblotting

Forty µg of hippocampal protein per sample was subjected to PAGE electrophoresis using 4–12% Bis-Tris Midi Gel (Invitrogen) and transferred to a blotting membrane with the iBlot system (Invitrogen). The membrane probed with Goat anti-CHAT (1∶1000, Millipore) was blocked with Western Blocker Solution (Sigma). All other membranes were blocked with 5% milk in TBS/1.5% Tween (TBS-T), washed with TBS-T, and probed overnight with either rabbit anti-p75NTR (1∶3000, Advanced Targeting Systems), mouse anti-GFAP (1∶1000, Cell Signaling Technology), rabbit anti-TrkA (1∶1000, Millipore), rabbit anti-DCX (1∶1000, Cell Signaling Technology), rat anti-ALK-1 (1∶1000, R & D Systems), rabbit anti-BMP9 (1∶1000, Abcam), or mouse anti-β-actin (1∶5000, Sigma). Following incubation with the primary antibody, blots were incubated in species-specific anti-IgG-HRP: anti-Rabbit-HRP (1∶4000, Bio-Rad), anti-Goat/Sheep-HRP (1∶2000; Sigma), or anti-mouse-HRP (1∶2000, Bio-Rad).

Reactive bands were detected with SuperSignal West Femto chemiluminescent substrate (Pierce, Rockford IL). Chemiluminescence was captured with a Kodak ImageStation 440CF and the band intensities were quantified with Kodak 1D Image Analysis software.

### RNA Extraction and Reverse Transcriptase PCR

Following the dissection of the basal forebrain, tissues were homogenized in buffer RLT (Qiagen) and frozen at −70°C. Total RNA was extracted from homogenized samples using an RNAeasy kit (Qiagen) according to manufacturer’s instructions. RNAs were used for reverse transcriptase PCR using Superscript™ One-Step RT-PCR with Platinum® *Taq* (Invitrogen Life Technologies). First strand cDNA synthesis was performed using the extracted total RNA (10 ng for β-actin, 25 ng for Chat, and 50 ng of RNA for Bmp9), oligo dT primer and reverse transcriptase at 48°C (45 min). Primers used for PCR include β-actin (Forward: CACAGCTGAGAGGGAAATC, Reverse: TCAGCAATGCCTGGGTAC), Chat (Forward: CGGGATCCTGCCTCATCTCTGGTGT, Reverse: GGCGGAATTCAATCACAACAT), and Bmp9 (Forward: TAAACCTCAGCGGCATTCC, Reverse: AAACGACCATGCTTCCTTCC). PCR was performed using Platinum *Taq* DNA polymerase with a denaturing step for 2 min at 94°C, followed by 32–40 cycles of 1 min at 94°C, 1 min at 58°C and 2 min at 72°C (32 cycles for β-actin, 36 for *Chat*, and 40 for *Bmp9*) and terminated by an elongation step at 72°C for 7 min. PCR products were displayed on a 10% polyacrylamide gel and stained with ethidium bromide. PCR products were visualized with Kodak Image Station 440 and product intensities were quantified using Kodak software.

### Immunohistochemistry

Brains were dissected and immediately fixed in 5 volumes of PLP fixative (4% paraformaldehyde, 75 mM lysine, 10 mM sodium periodate; pH 7.4) at 4°C overnight and cryoprotected using 10% and 20% glycerol/2% dimethylsulfoxide, in 0.1 M PBS, pH 7.3 (24 h each). Serial, frozen sections (40 µm, coronal) were cut with a sliding microtome from the anterior frontal pole of to the caudal occipital region. All sections intended to be subjects of comparative analyses were processed together and incubated for the same time periods in all of the reagents. For Aβ42 immunohistochemistry, sections were treated with>95% formic acid (Sigma) for 2 min with gentle agitation, washed with PBS, and then transferred to a solution of PBS/10% Goat Serum (Gibco) for 1 h at room temperature (RT). Sections were probed with rabbit anti-Aβ42 (1∶2500, Invitrogen) overnight at RT in 0.3% triton-X 100, 2% goat serum (Gibco), 0.008% sodium azide PBS. Following washing, sections were probed with goat anti-Rabbit-HRP (Millipore, 1∶1000) in a solution of 2% goat serum/PBS for 3 h at RT. After incubation in a developing solution containing diaminobenzidine (DAB), sodium imidazole, and hydrogen peroxide, sections were mounted on subbed slides. Photomicrographs were taken with 2X and 4X magnification objectives and images of the hippocampus were analyzed with ImageJ software (NIH).

### Microscopy, Fluorescence Imaging and Quantification of BFCN Markers

BFCN were imaged using fluorescence microscopy of GFP-expressing cells and by immunofluorescence following anti-75 antibody staining. For p75 immunofluorescence, free floating sections were incubated for 3 h in a blocking buffer consisting of 10% normal donkey serum and 0.3% Triton X-100 in PBS and subsequently overnight in 1% BSA, 0.3% Triton X-100 in PBS containing the rabbit anti-p75 polyclonal antibody (Cell Signaling Technology, 1∶3200). The sections were rinsed with PBS, blocked in the aforementioned blocking buffer for 3 h and incubated in the dark for 6 h with secondary Alexa Fluor-594 donkey anti-rabbit IgG antibody (Life Technologies, 1∶1000). After the final PBS rinse, the sections were mounted on on SuperfrostPlus slides (Fisher) allowed to dry at RT in the dark, coverslipped and stored at −20°C. Each PBS rinse step consisted of 3×10 min washes and all incubations were performed at RT on a rotating shaker. BFCN were imaged with Olympus IX81/DSU spinning disc confocal microscope. Exposure settings were adjusted using image acquisition software (IPLab v.4.0; BD Biosciences). These settings, including the exposure time, were kept constant in each detection channel for all sections imaged and no pixels read saturation. Subsequently these images were used to estimate average BFCN GFP fluorescence intensity and cell area with the NIH ImageJ software. All animals were used for this analysis (n = 6 per group) and all GFP-positive cells in each image were included.

Hippocampal sections were used to image immunofluorescence staining of DCX within the dentate gyrus. The protocol described above was followed except goat anti-DCX (1∶250, Santa Cruz) and secondary Alexa Fluor-594 donkey anti-goat IgG antibody (Life Technologies,1∶1000) were used instead.

### Data Analysis

Data for all experiments, presented as means ± SEM (n = 6), were analyzed by t-test or a one- or two-way ANOVA, as appropriate. *Post hoc* analyses were performed with a Fisher’s LSD test.

## Results

### IGF2 Infusion Reduces Hippocampal Amyloidosis in APP.PS1/Chgfp Mice

**Figure 1:**
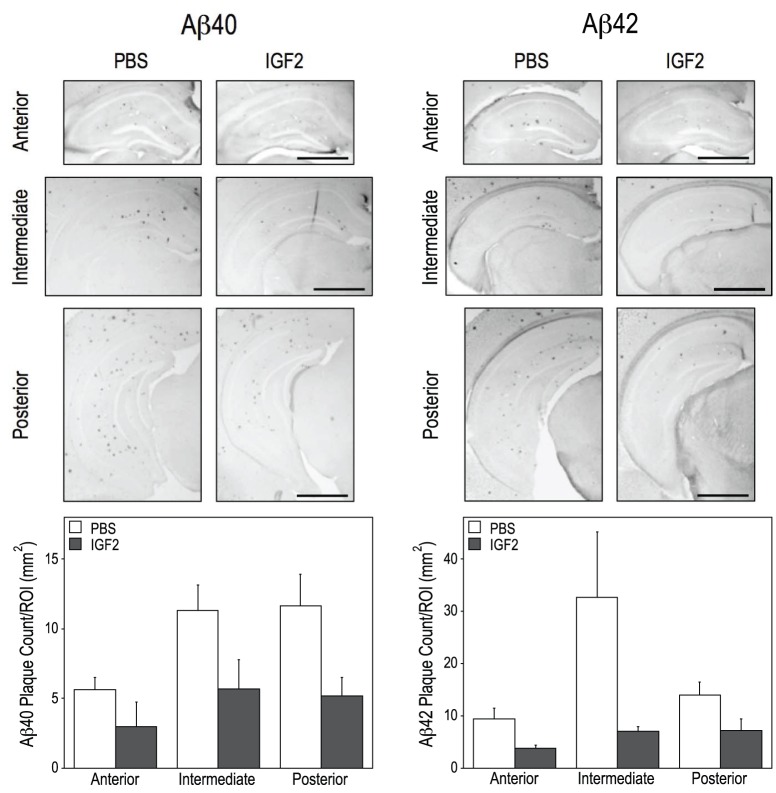
IGF2 infusion reduces the number of Aβ40 and Aβ42 plaques in the hippocampus. Immunohistochemistry for Aβ40 (left) and Aβ42 (right) was performed on anterior, intermediate and posterior hippocampal sections from 6-month old APP.PS1/CHGFP mice. Representative images from each treatment group are shown. The number of Aβ40 (left) and Aβ42 (right) plaques within each of the hippocampal sections was counted and means per group are presented for each region. IGF2 treatment significantly reduced the number of hippocampal plaques as determined by one-way ANOVA with repeated measures [A, F(1,10) = 6.987, p = 0.027; B, F(1,10) = 6.483, p = 0.029]. Scale bar represents 1 mm.

We assessed the amyloid plaque deposition in APP.PS1/CHGFP mice using Aβ40 and Aβ42 immunohistochemical staining and measuring plaque number per unit of tissue area in the anterior (bregma approximately −1.5 mm), intermediate (bregma approximately −2.4 mm) and posterior (bregma approximately −3 mm) hippocampus (Figure 1). The data were analyzed by a one-way ANOVA using the hippocampal sub-regions as a repeated measure. A 7-day infusion of IGF2 caused a significant reduction in the Aβ40- and Aβ42-positive plaque number (by 50–60%) (Figure1). We also measured the hippocampal levels of the solubilized Aβ40 and Aβ42 peptides using ELISA. There were no significant effects of IGF2 infusion on Aβ40 and Aβ42 levels by this method (data not shown).

### IGF2 Infusion Increases BFCN Cell Size in the Septum and Hippocampal CHAT Protein Levels

**Figure 2:**
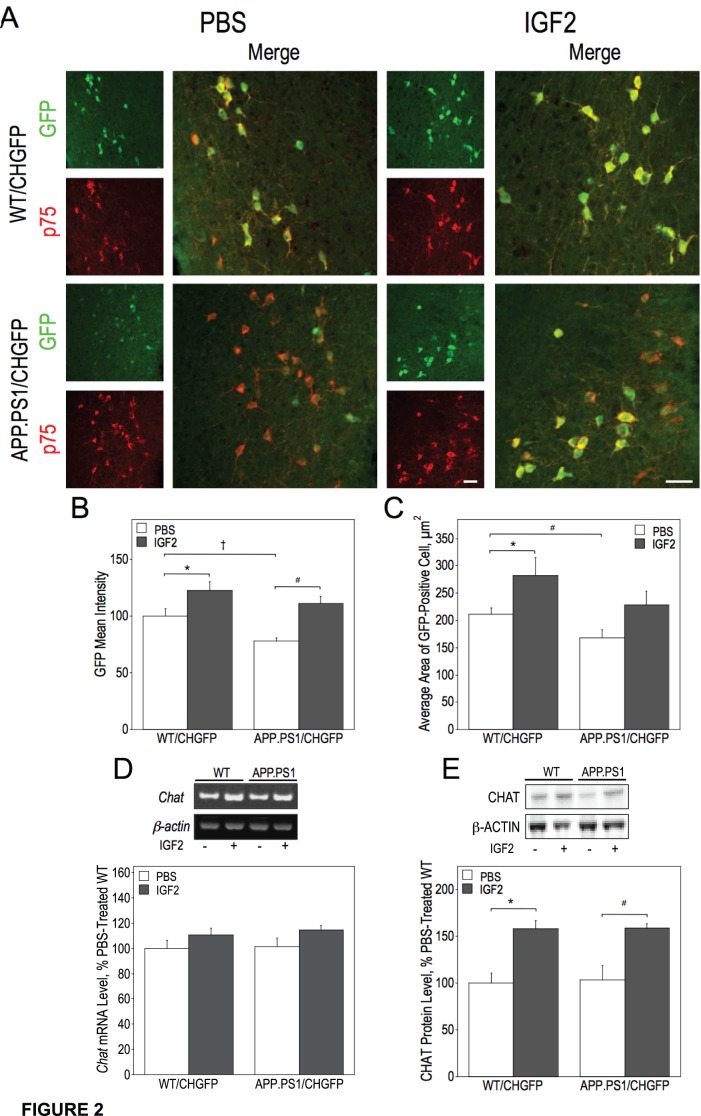
IGF2 infusion increases cell size of BFCN in the septum, septal *Chat* mRNA levels and hippocampal CHAT protein levels. Septal sections from WT/CHGFP and APP.PS1/CHGFP mice were stained with anti-p75NGFR antibody and imaged using a confocal microscope (A) and mean fluorescence intensity of GFP in BFCN (B) and average BFCN size calculated (C). Septal lysates were analyzed by RT-PCR to determine *Chat* mRNA levels (D). Hippocampal lysates were used to determine CHAT protein levels by immunoblot (E). Data were analyzed by two-way ANOVA followed by a post-hoc Fisher’s LSD test. GFP mean intensity was significantly affected by the infusion of IGF2 [F(1, 20) = 20.589, p = 0.002] and genotype [F(1, 20) = 7.294, p = 0.014]. Significant differences in GFP intensity between groups are indicated by * (p = 0.016), # (p = 0.001), and † (p = 0.022) (B, see brackets). The area of GFP-positive cells was also significantly influenced by IGF2 treatment [F(1, 20) = 8.770, p = 0.008] and genotype [F(1, 20) = 4.695, p = 0.043]. Significant differences in average area between groups are indicated by * (p = 0.014) and # (p = 0.035) (C, see brackets). IGF2 infusion also significantly increased the expression of CHAT mRNA [F(1, 20) = 5.120, p = 0.035] and protein [F(1, 20) = 29.956, p = 0.0001]. Significant differences in CHAT protein levels between groups are indicated by * (p = 0.004) and # (p = 0.006) (G, see brackets). Scale bar represents 50 µm.

To determine the effects of IGF2 on septal BFCN, we imaged these GFP-expressing cells in brain sections using fluorescence microscopy. To further verify the identity of these neurons we also stained the brain sections with an antibody to the BFCN protein marker, the low-affinity neurotrophin receptor, p75NGFR. As expected, septal BFCN expressed this protein (Figure 2A). We quantified the intensity of GFP fluorescence of BFCN as well as their average cell size. The intensity of BFCN fluorescence was reduced in untreated APP.PS1/CHGFP mice by 22% as compared to the WT/CHGFP mice (Figure 2B). IGF2 increased BFCN fluorescence intensity by 23% in the WT/CHGFP mice and by 35% in the APP.PS1/CHGFP mice (Figure 2B). Similarly, the average size of septal BFCN was reduced by 21% in APP.PS1/CHGFP mice as compared to WT/CHGFP mice and IGF2 increased the size of BFCN by approximately 30–34% in both the wild type and AD model mice (Figure 2C). In addition we found a small increase in the septal *Chat* mRNA levels in IGF2 infused mice of both genotypes as compared to untreated controls (Figure 2D). Hippocampal CHAT protein levels were similar in WT/CHGFP and APP.PS1/CHGFP mice and were increased by IGF2 infusion by approximately 50% in both WT/CHGFP and APP.PS1/CHGFP animals (Figure 2E).

### AD-like Pathophysiology and IGF2 Infusion Modulate the Expression of a Cholinergic Differentiating Factor, BMP9, and of Its Receptor, ALK1

**Figure 3:**
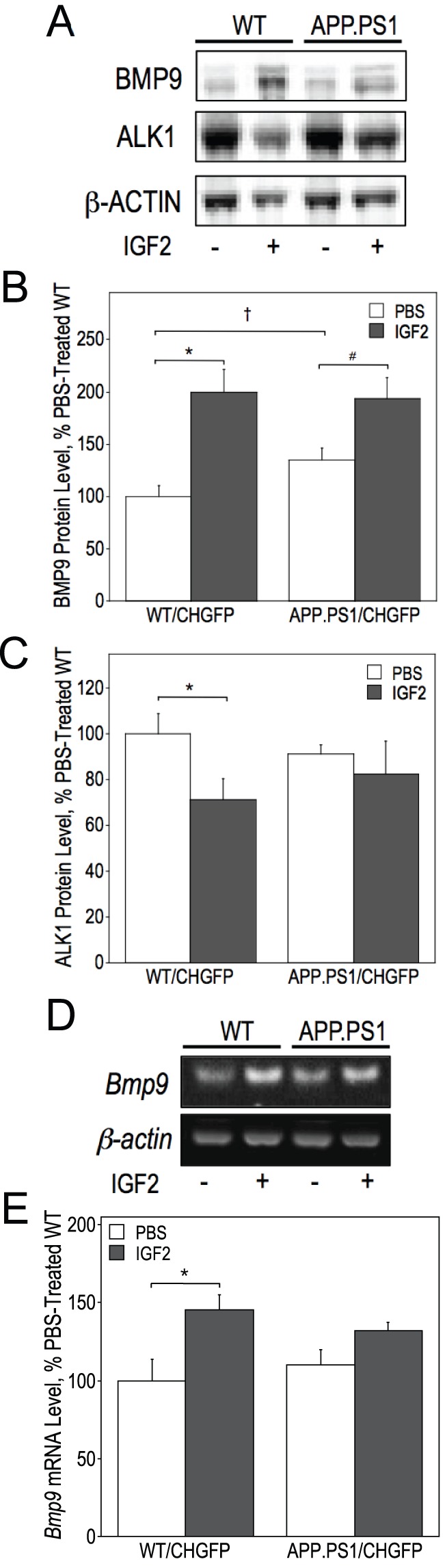
IGF2 infusion increases BMP9 expression and modulates the levels of its receptor, ALK1. Septal lysates from WT/CHGFP and APP.PS1/CHGFP mice were analyzed by RT-PCR to determine *Bmp9* mRNA levels (A,B). Hippocampal lysates were used to determine BMP9 and ALK1 protein levels by immunoblot (C,D,E). Data were analyzed by two-way ANOVA followed by a post-hoc Fisher’s LSD test. IGF2 infusion increased the expression of BMP9 mRNA levels within the septum [F(1, 20) = 12.885, p = 0.002]. Significant differences in BMP9 mRNA between groups are indicated by * (p = 0.003) (B, see bracket). BMP9 protein levels in the hippocampus were also significantly increased by the infusion of IGF2 [F(1, 20) = 21.770, p = 0.0002]. Significant differences in BMP9 protein level between groups are indicated by * (p = 0.001), # (p = 0.025), and † (p = 0.046) (D, see brackets). IGF2 treatment decreased the expression of ALK1 protein [F(1, 20) = 5.724, p = 0.026]. Significant differences in ALK1 protein level between groups are indicated by * (p = 0.031) (E, see bracket).

The acquisition of the cholinergic phenotype of BFCN during development [42]–[44] and its maintenance in adulthood [41] is controlled by bone morphogenetic protein 9 (BMP9, also known as growth/differentiation factor 2, GDF2). Therefore we measured the levels of BMP9 protein in the hippocampus in the control- and IGF2-infused mice. APP.PS1/CHGFP mice were characterized by a 40% increase in the basal levels of BMP9 protein relative to the WT/CHGFP animals and IGF2 infusion increased those levels in both mouse lines leading to similarly high BMP9 protein amounts in WT/CHGFP (2-fold vs controls) and APP.PS1/CHGFP mice (45% increase vs controls) (Figure 3A, B). BMP9 signals via its specific type I receptor, ALK1 [45]–[48] and we showed that ALK1 is expressed by BFCN [24]. We observed a reduction of ALK1 protein levels in the hippocampus of the WT/CHGFP mice infused with IGF2 (70% of controls). This effect of IGF2 was absent in APP.PS1/CHGFP mice (Figure 3A, C). Our previous studies showed that *Bmp9* mRNA is expressed in mouse septum [42]. We found that IGF2 increased *Bmp9* transcript expression in the septum of WT/CHGFP mice (by 47%) but this effect was not observed in APP.PS1/CHGFP mice (Figure 3D, E).

### IGF2 Infusion Increases the Levels of the Growth Factors in the Hippocampus

**Figure 4:**
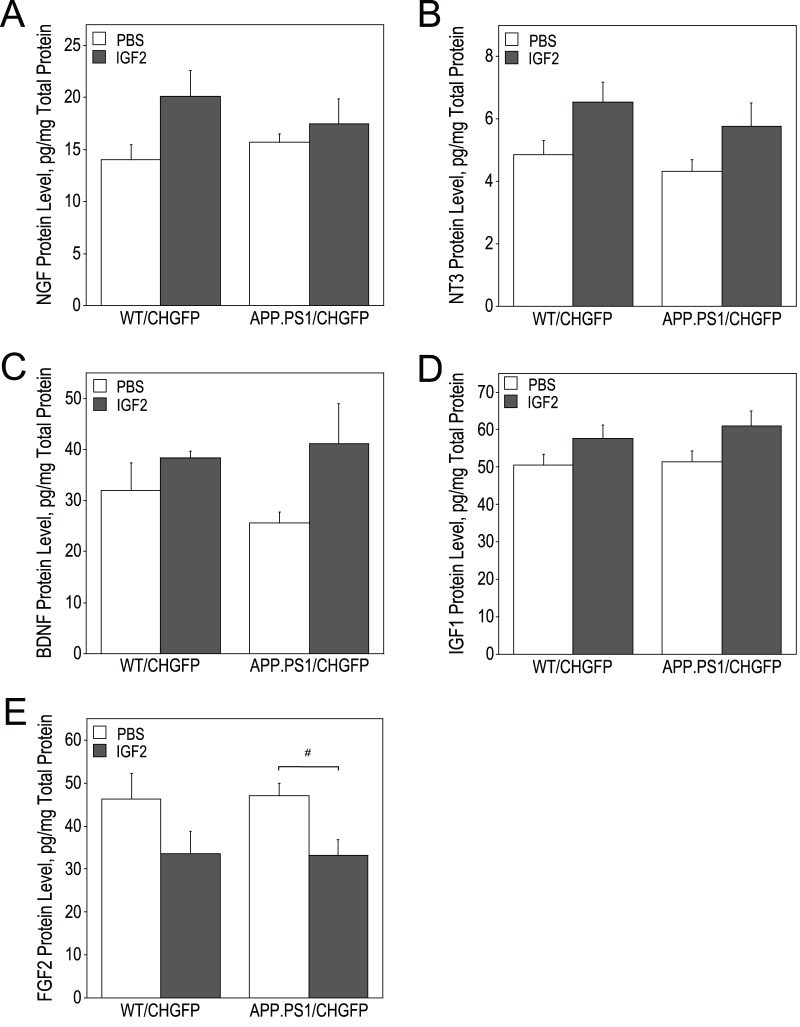
IGF2 infusion alters the level of NGF, NT3, BDNF, FGF2 and IGF1 in WT/CHGFP and APP.PS1/CHGFP mice. Hippocampal lysates were used to assay NGF (A), NT3 (B), BDNF (C), FGF2 (D) and IGF1 levels (E) by ELISA. IGF2 infusion increased the levels of all of these growth factors as determined by two-way ANOVA [NGF: F(1, 20) = 4.422, p = 0.047; NT3: F(1, 20) = 7.551, p = 0.012; BDNF: F(1, 20) = 6.373, p = 0.020; FGF2: F(1, 20) = 8.348, p = 0.009; and IGF1: F(1, 20) = 6.115, p = 0.022]. In addition, significant differences between groups are indicated by # (p = 0.047) (D, see bracket).

IGF2 infusion also modulated the expression of other proteins that are trophic to BFCN (Figure 4). We found a statistically significant overall increases in the levels of NGF, NT3, BDNF, and IGF1 in the hippocampus of IGF2-treated mice. In contrast IGF2 infusion significantly reduced hippocampal FGF2 levels (to 70% of controls in APP.PS1/CHGFP mice) (Figure 4E).

### IGF2 Infusion Increases Hippocampal Neurogenesis

**Figure 5:**
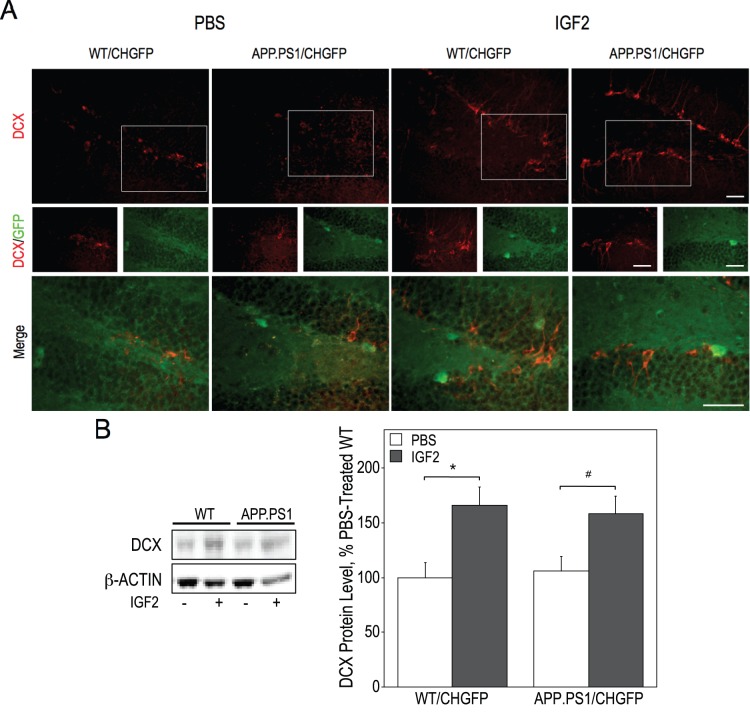
IGF2 infusion increases doublecortin (DCX) expression in the dentate gyrus. Hippocampal sections from WT/CHGFP and APP.PS1/CHGFP mice were stained with anti-DCX antibody and imaged using a confocal microscope (A). Hippocampal lysates were used to determine DCX protein levels by immunoblot (B). Data were analyzed by two-way ANOVA followed by a post-hoc Fisher’s LSD test. DCX protein levels were significantly increased by IGF2 infusion [F(1, 20) = 15.828, p = 0.001]. Significant differences between groups, as determined by a post-hoc Fisher’s LSD test, are indicated by * (p = 0.005) and # (p = 0.022) (see brackets). Scale bar represents 50 µm.

Previous studies showed that IGF2 stimulates adult hippocampal neurogenesis. We measured the levels of doublecortin (DCX, a marker of neuronal precursor cells and immature neurons) as an index of this process [49], [50]. Using confocal microscopy of hippocampal sections stained with an anti-DCX antibody, an increase in DCX-positive staining by IGF2 infusion was readily apparent (Figure 5A). We also found a 60% increase in the levels of DCX in the hippocampus of IGF2-infused wild type and AD-model mice as compared to controls using immunoblot analysis (Figure 5B).

### IGF2 Infusion Slightly Reduces Hippocampal Gliosis in APP.PS1/CHGFP Mice

**Figure 6:**
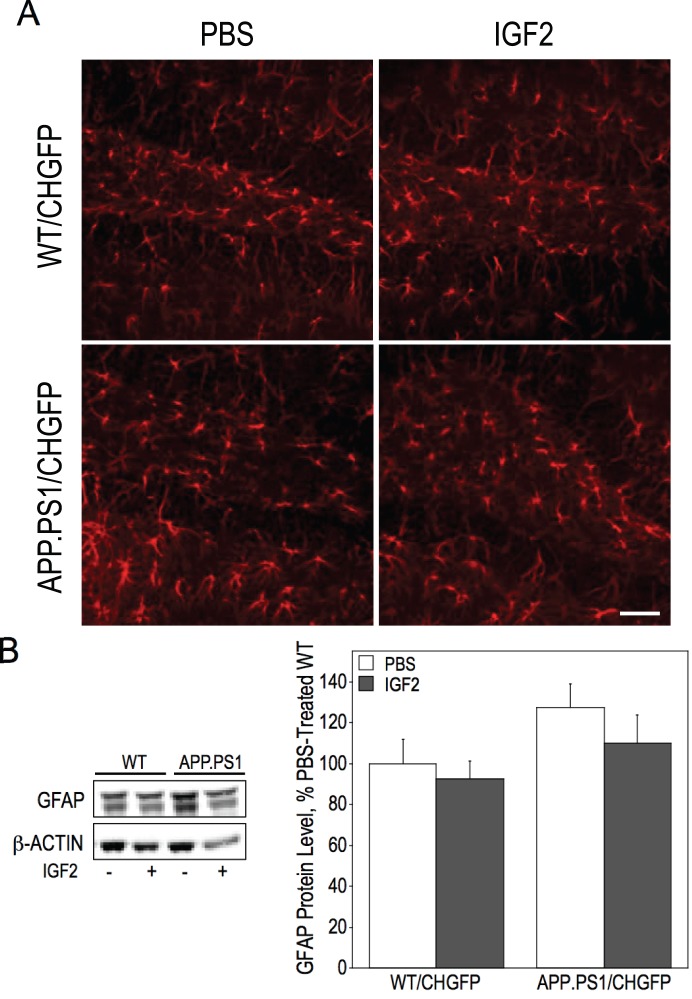
IGF2 infusion reduces GFAP protein levels in the hippocampus. (A) Representative images of GFAP immunofluorescence in the hippocampus of WT/CHGFP and APP.PS1/CHGFP mice using confocal microscopy. (B) GFAP protein levels were measured in hippocampal lysates by immunoblot. APP.PS1/CHGFP mice had significantly higher levels of GFAP than WT/GFP mice, as determined by two-way ANOVA [F(1, 20) = 4.590, p = 0.045]. Scale bar represents 50 µm.

APP.PS1 mice are reportedly characterized by hippocampal gliosis that increases with age as determined using GFAP immunostaining and qPCR assays [51], [52]. Using immunoblots, we found early signs of increased GFAP expression the hippocampus of APP.PS1/CHGFP mice (Figure 6). IGF2 infusion had no effect on GFAP levels.

## Discussion

The results show that administration of IGF2 to the brain of the AD model APP.PS1/CHGFP mice ameliorates the central pathophysiologic feature of AD, the accumulation of amyloid plaques in the hippocampus. This action of IGF2 was observed after only a one week infusion, consistent with the notion that plaque turnover can be rapid. Previous studies showed that plaque formation is exceptionally fast occurring within one day in APP.PS1 mice [53]. Thus, it is conceivable that IGF2 could inhibit the generation of new plaques, an effect that would manifest as fewer plaques in the IGF2-treated mice as compared to controls. Similarly, plaque burden may be reduced within days in APP.PS1 mice by certain drugs, e.g. PPARγ- agonists [54], indicating that if IGF2 acted by accelerating plaque clearance, this too could be observed within the time period of IGF2 administration.

We present novel observations that IGF2 administration increases hippocampal CHAT protein levels and *Chat* mRNA expression in the septum, and moreover, that the IGF2-treated mice are characterized by increased size of septal BFCN. The latter observation points to novel trophic actions of IGF2 on cholinergic neuronal morphology *in vivo*. Our findings related to modulation of CHAT levels by IGF2 are consistent with *in vitro* studies showing that IGF2 is a trophic factor for cultured BFCN as assessed by cell viability and CHAT expression [55]–[57]. Moreover, fetal IGF2-treated BFCN transplanted into the hippocampus exhibited higher long-term survival than control neurons [58]. IGF2 was equally as effective in elevating CHAT expression in WT/CHGFP- as in APP.PS1/CHGFP mice. These data indicate that the AD-model mice analyzed at 6 months of age, i.e. relatively early during the progression of the AD-like pathology, exhibit similar responsiveness to IGF2 as their wild type siblings.

The antiamyloidogenic action of IGF2 observed in APP.PS1/CHGFP mice may be the consequence of the increased cholinergic tone evoked by IGF2 because cholinergic neurotransmission activates α-secretase which hydrolyzes APP within the Aβ sequence precluding amyloid formation [59]. Indeed, in APP.PS1 mice subjected to a specific BFCN lesion, produced by the immunotoxin anti-p75NGFR-saporin, there is a rapid acceleration of amyloid plaque deposition in the hippocampus that can be appreciated within days [60], [61] indicating that normal cholinergic innervation slows down the generation of new plaques. Similarly, in AD model mice with deleted m1 muscarinic receptor, Aβ levels and amyloid plaque numbers are increased [62]. However, in contrast to the reduction of Aβ42 plaque density in the hippocampus of IGF2-infused mice, we found no effect of IGF2 on total Aβ40 and Aβ42 levels measured by ELISA in hippocampal extracts. Interestingly other reports also indicated relatively more robust effects of various treatments on plaque burden as compared to Aβ peptide levels in mouse models of AD [63]–[65]. One possible mechanism that could explain our observations might be that IGF2 slows down the aggregation and deposition of the Aβ peptides into plaques or, alternatively, that IGF2 promotes plaque clearance (e.g. by glial cells), involving transfer of the peptide from plaques to an intracellular compartment where it may be degraded. If this were the case, higher amounts of Aβ peptides would be present in non-plaque form in brains of IGF2-treated mice as compared to the vehicle-infused animals. Our ELISA method – which includes amyloid solubilization in guanidine hydrochloride – was designed to detect all Aβ42 (i.e., present in plaques, in diffuse extracellular amyloid and the intracellular peptide).

Our studies show that IGF2 infusion generates a trophic environment for BFCN by increasing the levels of several proteins that are neuroprotective for these neurons, indicating that IGF2 may not only act directly on BFCN but also, by creating a trophic milieu for these cells, supports their function via additional paracrine mechanisms. In particular, IGF2 dramatically increased hippocampal BMP9 protein levels in both the wild type and AD model mice. We have previously shown that BMP9 infused icv to mice with experimental injury to these neurons, prevents BFCN loss [41] and a 7-day icv infusion of BMP9 in APP.PS1/CHGFP mice reduces hippocampal and cortical amyloidosis and counteracts the cholinergic defect [66]. In contrast, IGF2 lowered the levels of the BMP9 receptor, ALK1, specifically in WT/CHGFP mice, possibly leading to desensitization/tolerance to the IGF2-induced BMP9 in these animals. In APP.PS1/CHGFP mice no such downregulation of ALK1 was observed suggesting that the BMP9 induced by IGF2 would be expected to signal productively in these mice. Moreover, hippocampal BMP9 levels were higher in APP.PS1/CHGFP mice than in WT/CHGFP controls, suggesting that the induction of BMP9 synthesis may be part of an adaptive response to the AD-like pathophysiologic process that occurs in APP.PS1/CHGFP mice. While there are no data on the levels of BMP9 in AD brain, the levels of a related protein, BMP6 (but not BMP2 and BMP7), are increased in the hippocampus of AD patients and in a mouse AD model [67]. Similarly the levels of BMP4 mRNA [68] and the number of BMP4-positive cells [69] are reportedly increased in the hippocampus of APP.PS1 mice.

IGF2 increased the hippocampal levels of NGF, BDNF, and NT3 to varying degrees in WT/CHGFP- and APP.PS1/CHGFP mice. These neurotrophins support the viability, the cholinergic phenotype, axonal growth, synaptogenesis, and function of BFCN. NGF, whose levels rose slightly in IGF2-treated mice, is a prototypic trophic factor for septal cholinergic neurons [70] whose therapeutic utility for AD has been explored [71] (http://clinicaltrials.gov/ct2/show/NCT00876863). In contrast to the small response of NGF levels to the IGF2 treatment, hippocampal levels of BDNF increased considerably (by 60%) in IGF2-infused APP.PS1/CHGFP mice, suggesting that some of the actions of IGF2 on BFCN could be mediated by the rise in BDNF. BDNF supports BFCN survival and elevates CHAT expression in cell culture [72]–[76]. Moreover, BDNF is necessary for postnatal maturation of BFCN i*n* *vivo* [77]. In rats, treatment with BDNF prevents axotomy-induced degeneration and loss of CHAT expression in BFCN [78], [79]. NT3 levels in the hippocampus were increased by 30% in IGF2-treated mice as compared to controls. This neurotrophin promotes the extension of cholinergic axons towards their hippocampal and cortical target neurons and facilitates cholinergic synapse formation on these cells [80]. Furthermore, NT3 is neuroprotective for cortical neurons cultured in the presence of Aβ and attenuates Aβ-mediated apoptosis of these cells [81].

Interestingly, purified BFCN from AD patients are characterized by reduced expression of mRNAs encoding the NGF receptor, TRKA, the BDNF receptor, TRKB, and the NT3 receptor, TRKC, as compared to matched control subjects [82], [83], suggesting that increased levels of the neurotrophin ligands for these receptors could functionally offset reductions in their levels and/or activity. Indeed, recent reports indicate that administration of a small molecule TRKB agonist [84] or BDNF delivery via a viral vector [85] reversed memory impairments seen transgenic AD model mice.

Previous studies showed that the levels of IGF1 tend to be reduced in plasma of AD patients [86] and that APP.PS1 mice, crossed with a strain engineered to have reduced circulating IGF1 levels, are characterized by high brain amyloid burden [87]. In contrast, administration of IGF1 reduces brain amyloidosis in AD model mice [88]. There is also evidence that IGF1 protects neurons against Aβ-induced toxicity in culture [89]. Thus, the modestly increased levels of IGF1 by IGF2 seen in APP.PS1/CHGFP mice may potentially help to slow down the AD-like pathophysiologic process in these animals.

IGF2 infusion decreased the levels of hippocampal FGF2 both in the wild type- and APP.PS1/CHGFP mice. These data are consistent with our previous studies showing that icv administration of BMP9 in mice reduces the hippocampal levels of FGF2 [41]. Perhaps, the increased expression of BMP9 evoked by IGF2 administration (see above) was responsible for the reductions of FGF2 levels. However, previous studies showed that in mouse models of AD, hippocampal expression of FGF2, mediated by a viral vector, improves memory function, enhances long-term potentiation in the CA1 region and reduces Aβ levels [90], indicating a potential for the use of FGF2 as a therapeutic agent for AD.

AD model mice, including the APP.PS1 mice, exhibit impaired adult hippocampal neurogenesis as they age [91]–[94]. In this study, the 6-month-old APP.PS1/CHGFP mice had similar expression of DCX as the wild type mice. Our data showing enhanced DCX expression evoked by IGF2 are consistent with previous reports that this growth factor upregulates neurogenesis [12], [13], [15].

IGF2 signals by activating several classes of receptors including the insulin receptor (IR), insulin-like growth factor receptor 1 (IGF1R) and IGF2R [95]. These proteins are expressed in brain in cell-specific manner [95] and mediate the IGF2 signal either via a tyrosine kinase cascade (IR, IGFR1) or via a G-protein (IGF2R) [96]. As noted above, IGF2 facilitates memory function. Depending on the type of memory examined these effects are mediated by IGF2R (inhibitory avoidance) [10], [11] or IGF1R (fear extinction) [12]. It will be interesting to determine which receptors mediate the multiple IGF2 actions reported here.

In conclusion we report that IGF2 is effective in reducing the amyloid burden, enhancing cholinergic function, and generating a neurotrophic milieu for cholinergic neurons in a model of AD, thus providing evidence that the IGF2 signaling pathway may constitute a novel therapeutic target in AD.
